# Computational pathology for musculoskeletal conditions using machine learning: advances, trends, and challenges

**DOI:** 10.1186/s13075-021-02716-3

**Published:** 2022-03-11

**Authors:** Maxwell A. Konnaris, Matthew Brendel, Mark Alan Fontana, Miguel Otero, Lionel B. Ivashkiv, Fei Wang, Richard D. Bell

**Affiliations:** 1 Research Institute, Hospital for Special Surgery, New York, USA; 2 Orthopedic Soft Tissue Research Program, Hospital for Special Surgery, New York, USA; 3 Department of Population Health Sciences, Weill Cornell Medical College, New York, USA; 4 Institute for Computational Biomedicine, Department of Physiology and Biophysics, Weill Cornell Medicine, New York, NY USA; 5 Center for Analytics, Modeling, & Performance, Hospital for Special Surgery, New York, USA; 6 Arthritis and Tissue Degeneration Program, Hospital for Special Surgery, New York, USA; 7 Rosenweig Genomics Center, Hospital for Special Surgery, New York, USA

**Keywords:** Computational pathology, Machine learning, Image analysis, Convolutional neural network, Deep learning, Histopathology, Orthopedics, Rheumatology

## Abstract

Histopathology is widely used to analyze clinical biopsy specimens and tissues from pre-clinical models of a variety of musculoskeletal conditions. Histological assessment relies on scoring systems that require expertise, time, and resources, which can lead to an analysis bottleneck. Recent advancements in digital imaging and image processing provide an opportunity to automate histological analyses by implementing advanced statistical models such as machine learning and deep learning, which would greatly benefit the musculoskeletal field. This review provides a high-level overview of machine learning applications, a general pipeline of tissue collection to model selection, and highlights the development of image analysis methods, including some machine learning applications, to solve musculoskeletal problems. We discuss the optimization steps for tissue processing, sectioning, staining, and imaging that are critical for the successful generalizability of an automated image analysis model. We also commenting on the considerations that should be taken into account during model selection and the considerable advances in the field of computer vision outside of histopathology, which can be leveraged for image analysis. Finally, we provide a historic perspective of the previously used histopathological image analysis applications for musculoskeletal diseases, and we contrast it with the advantages of implementing state-of-the-art computational pathology approaches. While some deep learning approaches have been used, there is a significant opportunity to expand the use of such approaches to solve musculoskeletal problems.

## Introduction

Histopathological examination of tissue biopsy specimens and surgical materials, first developed in the 17th century [1], remains an essential tool for disease diagnostics [2] and evaluation of pre-clinical models [3] in orthopedics and rheumatology. Utilizing thin sections of tissues stained with dyes that reveal key structures, such as hematoxylin and eosin dyes, examination of nuclei and cytoplasm/extracellular matrix can reveal pathologic tissue and cellular alterations. While histologic evaluation of tissue biopsy specimens by experts (i.e., pathologists) remains the gold standard, it is also an approach prone to tissue sampling and interpretive biases [4]. A common solution is to assemble a panel of experts to independently grade to consensus or average their scores [3]. This can lead to an analytical bottleneck that can be prohibitively costly or otherwise difficult to overcome given shortages of pathologists and their increasing clinical workload [5].

Machine learning (ML) methods, especially deep neural networks (DNNs), have gained immense popularity and improved performance since 2012 with the ImageNet competition, which showed that using large amounts of data to develop DNNs can improve performance [6]. DNNs have allowed for improvement in many computer vision-related tasks, such as image recognition and image segmentation. Additionally, with the advent and increased use of digital imaging, in particular whole slide imaging (WSI) [7], high throughput digitization of histologic slides provides access to the large datasets required by ML methods. The fields of orthopedics and rheumatology are appropriately positioned to capitalize on these breakthroughs [8]. In this review, we will discuss some general concepts of machine learning, outline a typical pipeline from tissue collection to model building (noting particular areas of concern), and discuss implementations of digital image analysis, including some using ML methods, to solve histologic image analysis problems of musculoskeletal (MSK) conditions.

### Overview of artificial intelligence and machine learning within the domain of image analysis

Artificial intelligence (AI) was conceived of in the 1940s as a way of mimicking human decision-making processes with computational programs [9] and ML is a subdiscipline of AI involving the process of building algorithms to learn patterns or rules from data. A neural network (NN) is a type of ML model that maps the input (e.g., clinical characteristics of a patient or pixel intensities of an image) to the output (e.g., whether or not a disease is present) through a series of non-linear transformations or functions. There are three distinct sections of a NN, the input layer, hidden layers, and output layer. The input layer contains the input data for a given sample (e.g., a patient’s clinical characteristics, an individual image). The hidden layers contain the transformations or functions of the network, and typically these transformations are organized into multiple layers within a given network, and thus these models are referred to as “deep” neural networks (DNNs) [10]. Lastly, the output layer contains the predictions of the network (e.g., will the patient live or die, does the image contain a chondrocyte or lymphocyte, does each individual pixel belong to the nucleus or not). These predictions are then compared against some standard, either a ground truth label in the supervised method (discussed later) or the original data itself, to generate prediction errors. An iterative optimization technique called backpropagation [11] is used to propagate the prediction errors through the model to update parameter values until there is no apparent improvement in the predictions, otherwise known as convergence. The quality of the learned model is highly dependent on the initial parameter of the functions, training data, and hyperparameters in the training procedure (reviewed in detail [8, 12]).

**Fig. 1:**
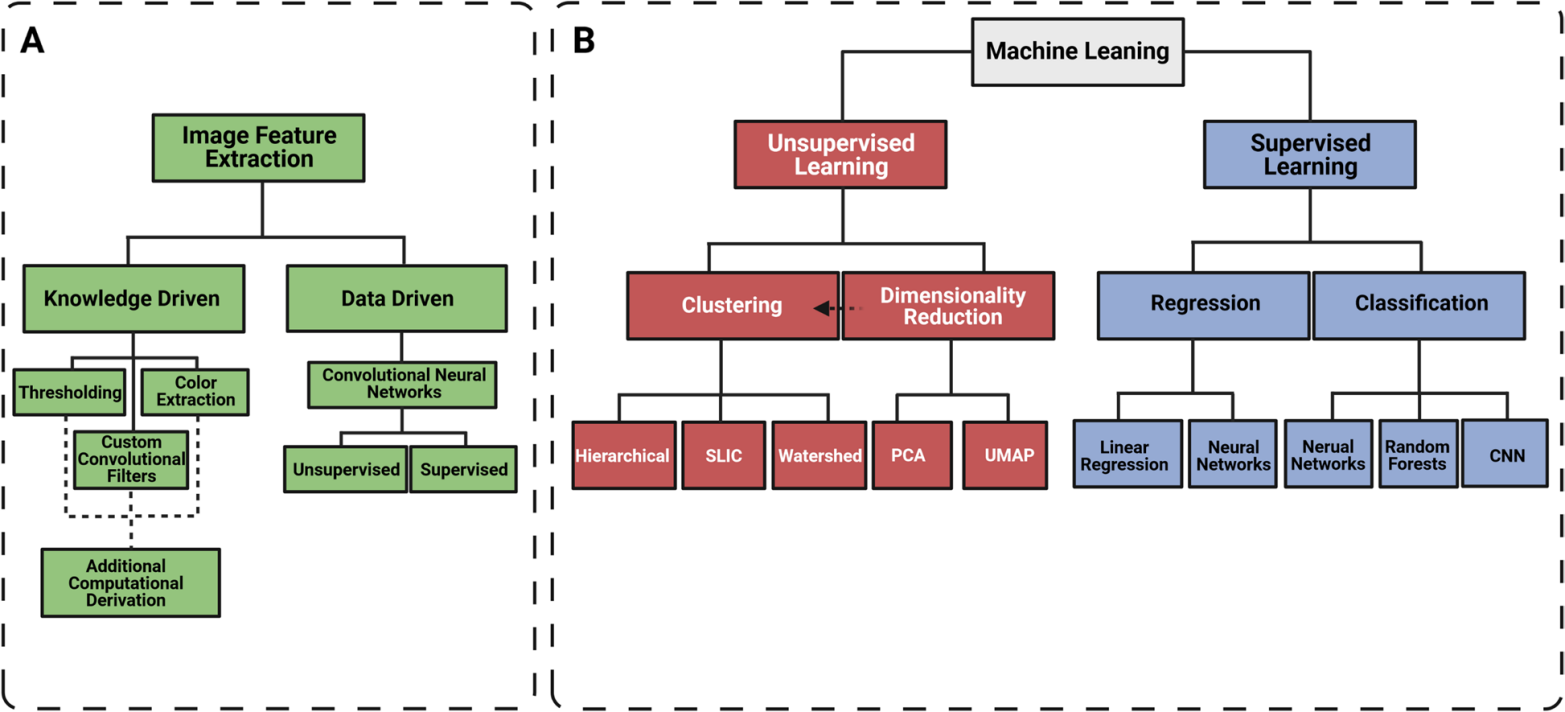
Overview of image analysis and machine learning subdisciplines. Extracting features is a critical step in image analysis and can be generalized to two methods, knowledge-driven and data-driven (**A**). Once image features are extracted, then one of two main ML subdisciplines are often used, (1) supervised learning and (2) unsupervised learning (**B**). A non-comprehensive summary of typical tasks solved with each subdiscipline, and common methods used to solve each task are listed below each subdiscipline. Solid lines indicate methodologies that follow the principles of each subdiscipline. Dotted arrows indicate direct links between the methods of disciplines

In the last 15 years, the applications of AI and ML models to attempt to solve healthcare problems in the domains of disease identification, prognosis, drug discovery, etc. have grown significantly [13]. In the field of medical image analysis, and more specifically histopathology or histomorphometry, ML can be implemented to improve efficiency and lower error rate of clinical tasks. For non-clinical tasks, ML can widen the analysis bottleneck by improving time to hypothesis testing and reducing the effort of expert pathologist to assess pre-clinical disease models. In the following, we will focus on the methods of extracting image features and the applications of two types of ML, i.e., supervised and unsupervised learning (Fig. 1), on these features for various downstream tasks.

#### Knowledge-driven vs data-driven feature extraction

**Fig. 2:**
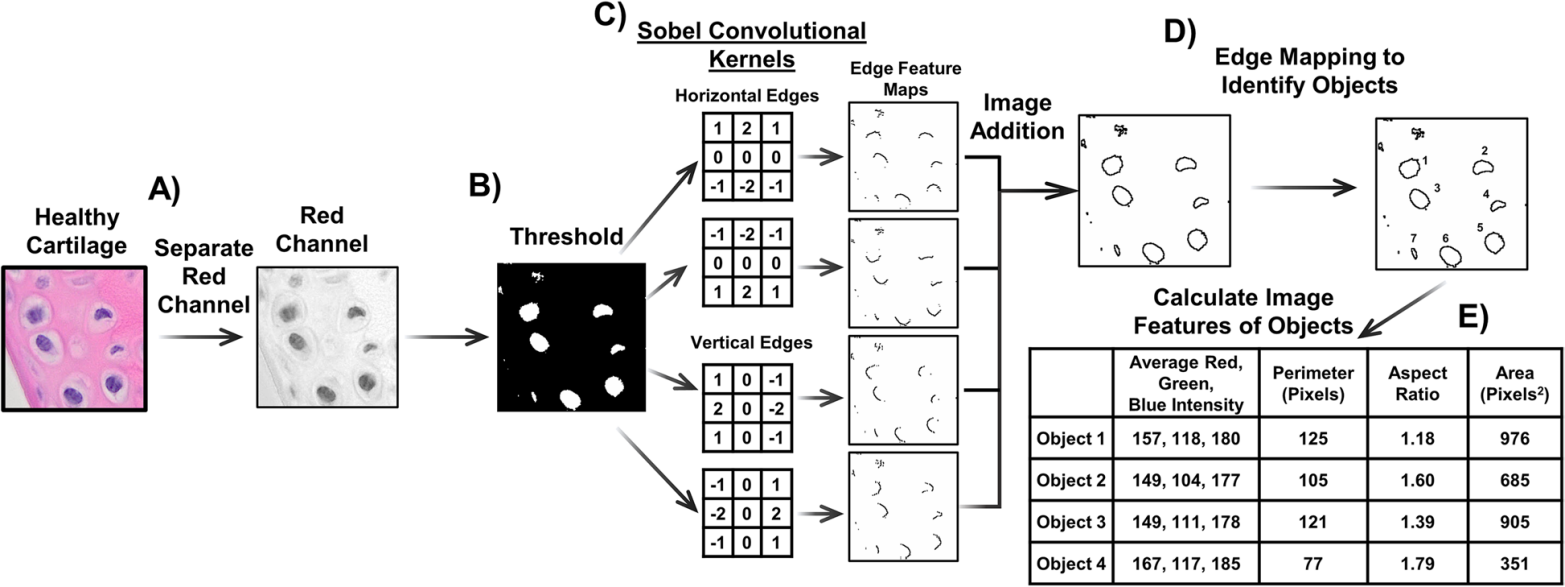
Examples of knowledge-driven feature extraction to identify nuclei. There are several mechanisms to extract information from an image. In this example, (**A**) the red channel of an image of H&E-stained cartilage with chondrocytes has been isolated and (**B**) thresholded to start the process of identifying the nuclei. (**C**) To identify the edges of the nuclei, convolutional kernels that have been designed to identify edges are applied (Sobel kernels) and the resulting images (feature maps) are added together. (**D**) Object detection algorithms, which can trace edges, can then be used to isolate the independent objects (nuclei) within the image. (**E**) Finally, color and shape features can be calculated to generate information about the nuclei that may help with pathologic analysis

**Fig. 3:**
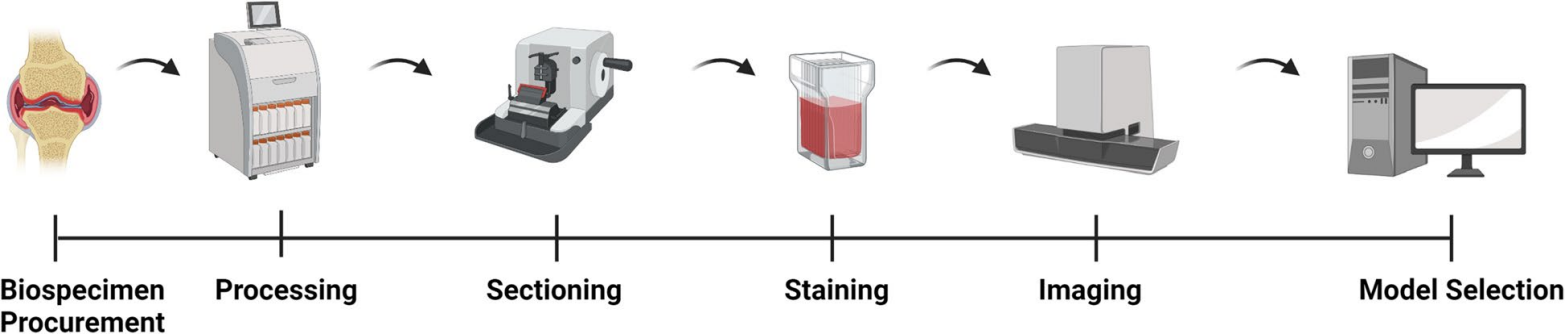
A generalized pipeline from tissue collection to model building. We have identified six main steps that are crucial in image analysis pipelines that can influence the results of an image analysis model: (1) biospecimen procurement, (2) processing, (3) sectioning, (4) staining, (5) imaging, (6) model selection

An image can be considered as a two-dimensional (gray) or three-dimensional (red, green, blue (RGB)) matrix, where each pixel can be represented by one number (gray) or three numbers (RGB) representing an intensity value. This can result in a high dimensionality of image features, especially since biomedical imaging data is taken at high resolution, which prevents the use of many standard machine learning approaches. Therefore, many approaches today work to generate meaningful lower dimensional feature representations of images, so that regression or classification techniques can be applied to this data type. There are typically two ways to reduce image feature dimensionality as demonstrated in Figs. 2 and 3: (1) knowledge-driven approach in which a priori knowledge of the image features and task to solve allows engineering of methods to extract meaningful data from the image and (2) data-driven approach in which self-sufficient feature extractors (e.g., data encoders) learn meaningful representations of the data. An example of a knowledge-driven approach to detect and measure nuclei is to extract the red pixel intensity on a H&E stained slide because it is inversely related to the nuclei (Fig. 2A). Image thresholding (Fig. 2B) to isolate high intensity vs low-intensity pixels and image filtering (Fig. 2C) are other knowledge-driven approaches which utilizes hand-crafted feature extractors. A 2D image filter, also called a convolutional filter or kernel, is a two-dimensional array of values and can be passed over an image to produce a new image, often called a feature map (Fig. 2C). For example, the Sobel filter or operator, invented in 1968 by Dr. Irwin Sobel and Dr. Gary Feldman [14], can identify horizontal and vertical edges and is used frequently in edge detection algorithms (Fig. 2C). There are many other variants of convolutional kernels that have been hand-crafted or empirically derived to detect other widely used image features [15]. Edges can be combined by image addition and object detection algorithms can be utilized to find objects, like nuclei (Fig. 2D). It may be important to know the color or shape parameters (e.g., RGB intensity, perimeter, aspect ratio, area) of an object to help describe some pathologic changes [16] (Fig. 2E). The benefit of these predefined features is that they can be based on characteristics that are already relevant for a given task based on deep understanding of the problem by domain experts. In addition, higher level or more detailed features can be extracted without the need for sophisticated models or large datasets to generate these features.

As an alternative to knowledge-driven feature extraction, deep learning models, such as convolutional neural networks (CNNs), can derive the important features for the predictive task of interest in a data-driven way [17]. CNNs are useful for image analysis because they maintain spatial information within an image using convolutional kernels, in contrast to standard neural networks which need to unroll the pixels of an image to form one long list of pixels removing the spatial information. These kernels are exactly like the image filters mentioned in the data-driven feature extraction setting, except that the values within the kernel can be altered during the learning process, specifically the backpropagation phase, of the network [18]. These kernels can be learned to extract different types of information, both low-level, such as color or edges at the beginning layers, as well as high-level features at the deeper layers within the model for the predictive task of interest. There have been various types of CNNs developed [6], many of which are available in the standard DL libraries and are ubiquitous in almost every biomedical image-based deep learning model.

Data augmentation is a method that can be used in both knowledge-driven and data-driven approaches to improve downstream model performance. As is the case with histology data, there are some transformations of the biomedical image that should not have an impact on model interpretation. Some examples include rotation, reflection, and scanner, reagent, or institutional shifts in the RGB color representation of the eosin and hematoxylin stains. To combat this, the model can introduce perturbations to the original image, such as including these rotations, reflections, and color shifts to increase the number of training examples, and to try to make the model more robust to these changes when applying ML or DL methods in practice. In addition, the color space of all images can be measured by investigating the RGB (or appropriate color maps) intensity and a normalization procedure can be performed attempting to control for unwanted color variation. Applications of these data augmentation steps will be discussed in subsequent sections.

#### Supervised learning

Supervised learning techniques are frequently used in image analysis [10, 19]. These approaches include two phases: training and testing. In the training phase, the ML model is provided with a dataset with input data samples (e.g., images) and their corresponding output targets (e.g., what class does the image belong to from a set of classes). The goal is for the model to learn how the inputs accurately map to the outputs. In the testing phase, we only have input samples whose targets are unknown, and the goal is to use the learned ML model to predict their corresponding targets. Depending on the types of the outputs, the supervised learning problem can be referred to as a classification (when the outputs are categorical or binary) or regression (when the outputs are ordinal or continuous) problems. Popular supervised learning problems for image analysis include image classification, which aims at assigning a label to an entire image (e.g., whether or not a knee joint has arthritis based on MRI), object recognition, which aims to judge whether a particular object is included in an image (e.g., whether or not the synovium is present within a section of histology tissue), or semantic segmentation, which aims to provide a classification label for every pixel in the image (e.g., draw a border around the synovial tissue in a histology section of a joint) [20].

#### Unsupervised learning

The second main type of ML technique is unsupervised learning, which uses algorithms to learn patterns from unlabeled data (i.e., no specific outcome or prediction target) to derive key insights about a dataset. Typical unsupervised learning problems include (1) clustering, which identifies coherent pixel groups in the image using techniques like watershed segmentation [21], simple linear iterative clustering (SLIC) superpixel segmentation [22], or coherent regions within the image using techniques like normalized cut [23] and (2) representation learning, which maps the original high-dimensional image features (e.g., pixels) to a new low-dimensional coordinate space, and the derived representations in the new space can optimally reconstruct the original image under certain reconstruction error measures. Principal coordinate analysis (PCA), Uniform Manifold Approximation and Projection (UMAP), and autoencoder are some popular representation learning techniques [24, 25]. Hierarchical clustering of image features or of the low-dimensional representations can also be leveraged to group similar/dissimilar objects within the image.

### A generalizable pipeline for image analysis approaches of histology with ML techniques

In order to make the ML models useful and practical for real-world MSK histology image analysis, we propose a generalizable pipeline including six steps from tissue collection to model selection that can influence final model performance (Fig. 3).

#### Biospecimen procurement

Specimen retrieval and tissue sampling from either pre-clinical models or in clinical settings is a critical first step that can greatly affect the generalizability of the model due to sampling bias, and the ability to precisely identify and diagnose meaningful pathological changes [26]. This is of particular importance when studying heterogeneous tissues, or in studies that address focal structural changes in relatively large joints, as shown in studies addressing the inability of a single biopsy specimen to capture solid tumor diversity [27] or in preclinical models of surgically-induced osteoarthritis (OA) that require extensive sampling across the joint to appropriately assess joint damage and disease development [3]. These concerns can sometimes be addressed with sectioning methods, discussed later, but gross dissection or biopsy techniques to acquire comprehensive sampling are first-line options. Thus, sampling consistency should be considered individually, maintained throughout the study, and reported carefully to ensure reproducibility and minimize sampling bias.

#### Processing

There are a plethora of cell culture and histology methods that can be used to generate an image for an ML model to analyze. Almost all methods utilize a fixation step to crosslink proteins preventing degradation, and while there is no standard fixation protocol (i.e., every tissue or cell requires some optimization), there are some common methods. Chemical fixation, such as aldehyde fixation, or precipitation methods, such as methanol, ethanol, or acetone, are widely used in MSK tissues. Time to fixation, length of fixation time, fixative agents, and the temperature of fixation may affect section quality and induce variability among samples [28]. Tissues are also frozen fresh or embedded in an aqueous cutting buffer to prepare for frozen sectioning. The time to freezing, length of time in a freezer, and the temperature that the sample is sectioned at can all affect the quality and stain consistency of the sections [29]. Musculoskeletal tissues are often decalcified and care should be taken to acknowledge how the pH of the tissue processing solutions potentially change the pH of the tissue because many stains are pH sensitive [30]. Having inconsistent processing that creates variable tissue artifacts within a study designed to utilize computational methods creates challenging problems for models to solve [31].

#### Sectioning

For tissue sectioning, the orientation and thickness should all be considered as potential confounding factors for downstream analysis [30]. For example, thicker sections may alter both staining quality, as well as the ability of the microscope to obtain a completely focused image. The latter is due to the depth of field of the objectives, which may be smaller than the section thickness. A 40× objective typically has a depth of field of 1 μm while tissue sections are commonly obtained between 5 and 10 μm, with some applications calling for much thicker sections. Orientation and anatomic location (i.e., histologic level) of the obtained sections have been well studied and standards are published in MSK disciplines [3]. While not formally studied in the fields of computational pathology, inconsistent orientation of the tissue as it is being sectioned may also impact the downstream image analysis. A model will likely either need curated data with known orientation or have sufficient training samples to learn the orientation variation in addition to what may be needed to understand pathology.

#### Staining

After sectioning, a stain must be applied to permit assessment of the tissues. Staining quality is often affected by the processing, fixation, and sectioning steps. Specific to the staining procedure, the pH of the reagents and the tissue, the staining time, reagent selection (e.g., bluing hematoxylin), the washing reagents and the number of washes can all alter the quality of the stain [28, 30]. These factors are both stain- and tissue-specific, and protocols must be standardized for each case, aiming to minimize staining artifacts and batch effects. The amount of time a slide sits on a shelf after staining was conducted can impact the stain quality, as many stains will oxidize over time. For long-term storage, a sealant should be applied to the edges of the cover slip, and it is advisable to acquire images for analysis shortly after staining to prevent such imaging artifacts. While no studies have directly compared the effects of various fixation, embedding, sectioning, or decalcification methods on ML models, any alteration from batch to batch of samples can be observed when the samples are stained as this represents a common bottleneck in all protocols [28]. These stain variation-driven artifacts result in batch effects that need to be accounted and corrected for either within or before model building.

Recent studies have addressed different approaches to deal with stain variation of tissue features. Otálora et al demonstrated that stain normalization, color augmentation, and a DL strategy called domain adversarial learning, a strategy that attempts to learn features independent of the domain (in this case the staining domain), both independently and when combined can improve the performance of CNNs [32]. They used two color heterogenous datasets which contained >25,000 H&E-stained breast and prostate cancer high power fields, which were annotated to indicate if they contained a mitotic figure or the Gleason pattern, respectively, obtained from multi-center repositories encompassing 20 different centers. In the first dataset, from the Tumor Proliferation Assessment Challenge, color augmentation with a standard CNN or the adversarial learning model when implemented independently performed similarly well (0.91–0.96 AUC), while in the second dataset, from The Cancer Genome Atlas (TGCA), color augmentation and adversarial learning when implemented together performed the best (0.69–0.77 AUC). Another study analyzed 13 different mitotic datasets with a standard CNN, varying only the stain normalization and color augmentation parameters [33]. Their results suggest that hematoxylin and eosin (H&E) color deconvolution with light augmentation and no stain normalization performs best. However, other color augmentation strategies including a style transfer inspired NN [34] or their own autoencoder stain normalization function performed only slightly worse. These strategies that evaluate stain and color augmentation suggest that including color augmentation in the ML pipeline is typically successful and depending on the image set, strategies that incorporate NN-based stain normalization or adversarial learning may improve performance. However, this is still an open area of research that needs further investigation.

#### Imaging

There are many available tools to digitize images, and the methods used to digitize a slide should be carefully considered for optimal downstream applications [35]. The selected magnification weighs heavily on many downstream procedures. For example, cellular or subcellular analyses may require 40× or higher magnification [36], while lower magnification may be sufficient for tissue-level analyses (i.e., segmentation). High magnification also requires more imaging time, larger storage resources, as well as significant computational capabilities if utilizing a DL approach. If a slide scanner is chosen to digitize the slides, scanner-to-scanner variation needs to be considered. A study of feature instability (e.g., variation in stain intensity, object shapes, object orientation relative to other objects) in the TGCA dataset found high levels of feature instability from the same slide imaged on different scanners, especially for the shape and stain intensity features [37]. This suggests that either the scanner should remain the same within a study, that sufficient normalization or augmentation approaches should be used to adjust the feature variance or sufficient training data be acquired to learn the feature variance. Potential sources of variation between scanners are the light source (e.g., type of bulb or light emitting diode (LEDs)), light path (type and number of objectives and their numerical aperture, number of mirrors, and condensers), the detector (charged couple device (CCD), complementary metal-oxide-semiconductor (CMOS), photomultiplier tube (PMT)), and the software (auto-focus algorithm, white balance/color balance algorithm, tile or line stitching algorithms). Advances in scanning software such as Deep Focus [38] that use DL to automatically detect of out-of-focus regions while scanning slides can immediately initiate a rescan, and improve image quality and the usability of data.

#### Model selection

Once images are acquired, selection of an appropriate image analysis model is a critical step in the pipeline. The complexity of the model should reflect the complexity of the problem. For example, segmenting 3,3′-diaminobenzidine (DAB)-stained tissue from the white background of a slide requires a relatively simple model, not considered to be a ML approach, such as evaluating the optical density of a pixel or region and thresholding [39]. In contrast, to detect a small piece of malignant tissue in a large tissue biopsy specimen, a DCNN model may need to be implemented [40]. The task also should be defined into one of the two domains, supervised or unsupervised learning (Fig. 1), to refine the techniques used. This decision-making process has been extensively reviewed elsewhere [8, 19, 41] and revolves around a critical concept: the amount and quality of labeled (annotated) data. If there is an abundance of high-quality labeled data, then a supervised strategy could be implemented to solve regression, classification, or segmentation tasks. For example, Graham and colleagues leveraged multiple datasets with over 75,000 annotated nuclei to build a DCNN for segmenting and classifying nuclei [42]; and the aforementioned example of malignant tissue classification utilized >20,000 WSI of biopsy specimens labeled as either malignant or benign [40]. However, different tasks may require less data and there are examples of models performing well using smaller datasets [43–46]. Importantly, these models need to be evaluated. When ground truth labels are available, performance metrics obtained by comparing the prediction with the labels, known as a confusion matrix, and subsequent calculations (e.g., sensitivity, specificity, F1) should be obtained. It is important to note that, while these performance metrics are analogous to the inter- or intra- rater reliability for the gold standard scoring systems, they are dependent on other sets of biases, such as the quality and amount of labeled data [4, 8, 13].

As an alternative, unsupervised methods, like watershed and SLIC, can perform image segmentation and when coupled with feature extraction methods (Fig. 2), dimensionality reduction, and/or clustering to describe the feature variation, there may be associations with pathology [47–49]. Since these methods are unsupervised, careful review of the results is needed and, if possible, should be validated with other measured outcomes. In addition, the modeler needs to decide if knowledge-driven or data-driven approaches are more suited to the analysis task (Fig. 2). For example, if the task is to segment tissue with low extracellular matrix (ECM) to cell ratio vs high ECM to cell tissue (i.e., fatty tissue vs bone), feature extraction of eosin stain intensity with a low training sample size and relatively simple model may be suitable. However, if the task is to segment cortical bone from trabecular bone, which is a substantially more difficult problem to solve, a DCNN may be required (Bell RD and co-authors, unpublished work). If a DL model is the chosen model, an excellent way to improve the training procedure of deep models is to utilize a technique called transfer learning. This transfers the pre-trained weights (e.g., values) of the convolutional kernels from another model and is useful because these kernels have already been trained to identify some image features that may overlap with image features in the task at hand [50]. Another way to improve training on small datasets is to perform image augmentation as mentioned previously [51].

### Computational pathology in musculoskeletal disciplines

There are more than 120 ML-based medical devices currently approved for medical imaging technologies by the US Food and Drug Administration (FDA) at this time (December 2021) [52], but only a few ML models are approved thus far for histopathological applications (e.g. cytology screening or cell classification) [41]. In research settings, ML has successfully been implemented to study chromatin distribution [53], subcellular organelles [36], identify generic nuclei [42], identify mitotic nuclei [54, 55], or to classify and segment various cancers types and associations with clinical outcomes [40, 56–58]. While ML is being increasingly used in clinical pathology, especially in cancer, it has not yet been extensively applied in the field of orthopedic and rheumatologic histopathology. Here, we provide a historical perspective of the steadily increasing amount of computational pathology applications in MSK conditions over the last 20 years, separated by tissue type (Table 1).

**Table 1:** Summary of Relevant Computational Pathology Work in Musculoskeletal Research

Tissue Type	Author/Year	Aim/Objective	Species	Stain	Imaging Modality	ML/feature Extraction Type	Technique/Model	Transfer Learning	Biological Specimens	Images (***N***)	Magnification	Performance Reported
Synovial Tissues	Kraan 2000 [39]	Quantification of CD3 and CD68+ cells	Human	IHC/DAB	Microscope, Camera	Knowledge Driven	Thresholding	No	9 RA, 5 Control subjects	70 (*n*=5/section)	40×	DIA Significantly correlated with manual cell counts, Spearman *ρ*: 0.56–0.95
	Haringman 2005 [59]	Quantification of CD68+ cells	Human	IHC/DAB	Microscope, Camera	Knowledge Driven	Thresholding	No	88 subjects (*n*=176 samples)	NR	40x	Validated in [39]
	Rooney 2007 [60]	Quantification of CD3 and CD68+ cells	Human	IHC/DAB	Microscope, Camera	Knowledge Driven	Thresholding	No	12 subjects (*n*≥6 samples/subject, *n* > 72)	24 tissue sections (*n*=12 slides)	1392×1040 pixel/image	ICC across sites: CD3+ 0.79; CD68+ 0.58; Spearman *ρ* Manual counts vs DIA : 0.62–0.98
	Morawietz 2008 [61]	Quantification of synovial features to validate synovitis score (enlargement of synovial lining (thickness), density of synovial stroma and inflammatory infiltrate (count))	Human	H&E	Microscope, Camera	Knowledge Driven	Thresholding	No	71 subjects (OA, *n*=22, PsA, *n*=7, RA, *n*=35, control, *n*=7)	NR	NR 584×720 pixel/image	Significant agreement in all measurements between the model and three independent pathology graders, Spearman *ρ*: 0.458–0.921
	Bell 2019 [62, 63]	Nuclear and cytoplasmic/ECM area	Mouse	H&E	Slide Scanner	Supervised, Knowledge Driven	Bayesian Classifier	No	NA	NA	40×	Previously Validated [75]
	Venerito 2021 [43]	Quantification and classification of synovitis	Human	H&E	Microscope, Camera	Supervised, Data Driven	CNN/Resnet34	Yes	12 subjects	150	4–20×	Validation Set - Acc.: 0.9; Prec.: 0.93; Rec.: 0.875 Test Set – Acc.: 1.0; Prec.: 1.0, Rec.: 1.0
Cartilage	Knight 2001 [64]	Vimentin and microtubule spatial organization	Bovine	IHC-IF	Confocal	Knowledge driven	Convolutional Filters	No	NR	NR	60×	Not validated
	Moussavi-Harami 2009 [65]	Automated and Objective implementation of the Mankin Scoring Scale	Human	Safranin-O	Microscope, Camera	Knowledge Driven	Custom Features Extraction	No	18 subjects (femoral heads, *n* =12, femoral condyles, *n*=5, tibial plateau, *n*=7)	NR	4× stitched (743,028 pixels/mm^2^ resolution)	Correlated well with Manikin Scoring (*r* ^2^=0.748)
	Yang 2019 [44]	Chondrocyte detection, count, and boundary segmentation	Rabbit	Safranin-O	Microscope, Camera	Supervised, Data Driven	CNN/U-Net	No	NR	260	256×256 pixel/image 0.32 μm/pixel	F1 scores: 0.86–0.90; segmentation accuracy: IoU=0.828; counted fewer chondrocytes than expert observer (*p*<0.001 paired *t* test)
Skeletal muscle	Klemencic 1998 [66]	Fiber Geometry	Human	Myofibrillar ATPase Activity	Microscope, Camera	Unsupervised, Knowledge Driven	Active Contour Model	NA	1 subject	NR	512×360 pixel/image 2.2 μm/pixel	Qualitative 92% correct by expert graders
	Kim 2007 [48]	Fiber geometry	Human	H&E	Microscope, Camera	Unsupervised, Knowledge Driven	Active Contour Model	NA	5 subjects	30	20× 640×480 pixel/image	663/679 (98%) fibers correctly detected;
	Sertel 2011 [67]	Fiber Geometry and Type	Rat	ATPase Activity	Microscope, Camera	Unsupervised, Knowledge Driven	Ridge detection	NA	12 subjects	25	10× 1280×1024 pixel/image	Overlap score: 91.3 ± 4.8%
	Liu 2013 [68]	Fiber geometry, Type, Myonuclei Counting	Mouse	IHC-IF	Microscope, Camera	Unsupervised/Supervised, Knowledge Driven	Ridge detection, SVM	NA	NR	20	20×	CSA Avg Diff: 0.88% Fiber type Avg Diff: 0.09% Nuclei counting Diff: 8.61%
	Smith and Barton 2014 [69]	Fiber Geometry, Type, MHC, Capillary Density, and CNF	Mouse	IHC-IF	Microscope, Camera	Knowledge Driven	Filtering and Watershed	No	8 subjects (*n*=4/group)	NR	NR	Difference Reported to Legacy Method (Simple Thresholding) CSA: 21.7% Fiber type: 7/177 fibers CNF: 9%
	Wen 2018 [49]	Fiber Geometry, Type, and Myonuclei Counting	Mouse	IHC-IF	Microscope, Camera	Semi-supervised, Knowledge Driven	Watershed with Euclidean Distance K-Means Optimization	No	16 (*n*=4/group)	NR	20×	Accuracy of ≥94% for fiber number, fiber type distribution, fiber CSA, and myonuclear number
	Miazaki 2015 [70]	Fiber Number and Geometry	Mouse	IHC-IF	Microscope, Camera—Stitched Into Mosaic	Unsupervised, Knowledge Driven	Filtering, Thresholding and Post-Hoc Shape Filtering	No	6 subjects (*n*=3/group)	6 20–30 stitched/sample	800×600 pixels/image 0.7 μm/pixel	NR
	Mayeuf-Louchart 2018 [71]	Fiber Number, Geometry, Type, CNF, Satellite Cells, and Vessel	Mouse	IHC-IF	Slide Scanner	Knowledge Driven	Filtering, Thresholding and Post-Hoc Shape Filtering	No	9 subjects (*n*=5, injured, *n*=4, control)	NR	20–40× 0.325–0.380 μm/pixel	No significant difference between expert graders and digital analysis in both uninjured and injured for all parameters, Mann-Whitney test *p* value: 0.4–0.7
	Reyes-Fernandez 2019 [72]	Fiber Number and Geometry	Human	IHC-IF	Microscope, Camera	Knowledge Driven	Filtering and Thresholding	No	57 subjects	NR	10× 9300×9900 pixels/image	Overall detection/segmentation of 89.3% of the total fibers (342/3212 not detected fibers across 10 samples analyzed); < 1% of the fibers misclassified (21/3212)
	Kastenschmidt 2019 [45]	Fiber Number, Geometry, Type, and CNF	Human and Mouse	IHC-IF	Microscope, Camera—Stitched into Mosaic	Supervised, Knowledge Driven	Filtering and Thresholding; SVM	No	NR (Human) 108 subjects (Mouse)	NR (Human) NR (Mouse)	10× (Human) 20× (Mouse) 1920×1440 pixels/image (Mouse)	Fiber number Acc.: 80–98%; CSA Acc.: 90–98%; CNF Acc.: 85–95%; Fiber Type Acc.: NR
	Encarnacion-Rivera 2020 [73]	Fiber Number, Geometry and Type	Mouse	IHC-IF	Microscope, Camera—Stitched into Mosaic	Knowledge Driven	Convolutional Filtering; Random Forest; Thresholding	No	32 subjects (*n*=29, C57BL/6J, *n*=3, mdx-4Cv)	~192 6/subject	10×	Count: *r* ^2^=0.99 with manual count CSA: Not Different than Manual annotation (2 annotators) Type: 1–5% False Positives
Other	Zhang 2016 [79]	Bone Fracture Healing Tissue Areas: New Cartilage, New Bone, New Fibrous Tissue, Bone Marrow and New Osteoblastic Area	Mouse	H&E – Orange G - Alcian Blue	Slide scanner	Knowledge Driven	Model Not Reported; Post-Hoc Area and Shape Adjustments	No	5 subjects (Mouse)	5	40×	ICCs between the Algorithm and Hand Drawn Areas: New Cartilage = 0.98, New Bone = 0.99, New Fibrous Tissue = 0.97
	Xia 2021 [46]	Wound Healing via Area of Primary Granulation, Secondary Granulation and Chondrogenic Tissue over Time	Mouse	H&E	Slide scanner	Supervised, Knowledge Driven	Random Forest	No	4 subjects (Mouse)	4	40×	Good agreement between model and pathologist scores
	Correia 2020 [47]	Develop DL-based score to mimic mRSS which discriminates SSc from normal skin	Human	Masson’s Trichrome	Slide Scanner	Unsupervised, Supervised, Data Driven	DCNN (Encoder of AlexNet); Principal Component Analysis; Logistic Regression	Yes	92 subjects, 168 biopsies; Primary cohort (*n* = 6 subjects, 26 SSc biopsies); Secondary cohort (*n* = 60 SSc and 16 controls, 148 biopsies)	100 randomly selected; Primary cohort (2600 image patches grouped by biopsy); Secondary cohort (7600 image patches grouped by biopsy)	40×	Primary Cohort Biopsy Score Correlation with mRSS: *R*=0.55, *p*=0.01; Secondary Cohort Diagnostic Score Logistic Regression to Classify SSc from Healthy (0.5 cutoff): AUC = 0.99 Misclassification rate = 1.9% (training), 6.6% (test); Secondary Cohort Fibrosis Score significantly correlated with mRSS: *R*=0.70 (training), 0.55 (test)

Abbreviations: *DAB* 3,3′-Diaminobenzidine, *AUC* area under the curve, *Avg* average, *CNF* centrally nucleated fibers, *CSA* cross-sectional area, *DCNN* deep convolutional neural network, *DL* deep learning, *Diff* difference, *DIA* digital image analysis, *ECM* extracellular matrix, *H&E* hematoxylin and eosin, *IHC* immunohistochemistry, *IF* immunofluorescence, *IoU* intersection over union, *ICC* intraclass correlation coefficient, *ML* machine learning, *mRSS* modified Rodnan skin score, *MHC* myosin heavy chain, *NA* not applicable, *NR* not reported, *OA* osteoarthritis, *RA* rheumatoid arthritis, *SSc* systemic sclerosis, *SVM* support vector machine

#### Synovium

Seminal work in the field of computer-assisted quantification of synovial features utilized thresholds of the RGB values in both H&E- and DAB-stained slides [39, 59, 60]. Utilizing this approach, the CD3 or CD64 DAB-positive tissue area correlated well with manually-counted positive cells [39]. Thresholding was also used to segment lymphocyte nuclei and quantify synovial thickness on H&E-stained tissue sections, which correlated with clinical scores with reasonable success in synovial biopsies from patients with rheumatoid arthritis, osteoarthritis, and psoriatic arthritis [61]. Using thresholding techniques on stained tissue is still a common approach used in both clinical and preclinical settings [74] and can be a reliable measurement of tissue features, but it is highly sensitive to staining variation or other batch effects. In a similar approach, a classifier trained to segment nuclear and cytoplasmic/extracellular matrix (ECM) area (naïve Bayes model implemented in Visiopharm [75]) was used to segment these areas in H&E-stained tissues from a murine model of inflammatory arthritis, which corresponded to histopathology scores [62, 63]. All the above studies used external methods of validation (i.e., correlation with other outcomes) and are limited by a lack of internal model performance validation (i.e., generating ground truth labels to test sensitivity and specificity of the segmentation). Venerito et al. used a CNN architecture (Resnet34) with transfer learning from the ImageNet dataset as one of the first applications of DL in synovial tissue analysis, training a classifier to discriminate between low- and high-grade synovitis of 150 synovial photomicrographs from 12 patients who underwent ultrasound-guided synovial tissue biopsy specimens [43]. The authors were able to correctly classify all 30 of the images as either low-grade or high-grade synovitis in the hold-out test set. However, more data would be needed to assess the real-world performance and generalizability of this model.

#### Cartilage

One of the first image analysis models to assess articular cartilage image features applied convolutional filters (horizontal and vertical Sobel filters) to confocal fluorescent images of chondrocytes stained with anti-β tubulin, anti-vimentin, and phalloidin. These feature maps were then thresholded to identify edges within the image to understand cytoskeletal arrangement of chondrocytes in agarose gel suspension culture over time [64]. Another effort to quantify cartilage biology with image analysis attempted to mimic the Mankin score [76]. Moussavi-Harami and co-authors modeled the articular surface using quadratic curves, measured depth of clefs nominal curve, and applied thresholding, edge detection, and convolutional filtering to estimate proteoglycan content, nuclei density, and blood vessel penetration past the tidemark. These computational approaches were validated by correlating with human quantified Mankin scores (*r* ^2^=0.78) [65]. Utilizing a DL approach, Yang et al. detected, counted, and segmented chondrocytes in Safranin-O stained slides of cartilage sections from an anterior cruciate ligament transection surgical rabbit model [44]. Training and validation images (256 × 256 pixels, *n* = 235 and *n*= 25, respectively) were hand-annotated by one expert and a U-NET architecture was trained, while 5 independent observers separately annotated an additional 35 images as an external test set. Internally, the model performed well with a IoU of 0.82; however, when compared to the 5 expert annotators, the model consistently predicted fewer chondrocytes and had poor IoU scores, suggesting a lack of generalizability from insufficient training data and/or too complex a model. Despite these limitations, this is the first work to utilize a DL architecture in chondrocyte segmentation, which begs for more exploration from the field.

#### Skeletal muscle

Skeletal muscle has had by far the most development of image analysis models within the MSK field. This is in part because muscle fiber geometry (e.g., cross-sectional area, perimeter, circularity, diameter) is an essential outcome of the histopathology assessment, thus providing a strong incentive to develop models. In addition, both basic stains, like H&E, and immunohistochemical stains provide high contrast imaging of the fiber edges, and edge detection algorithms are a mature field within image analysis, with models to specifically estimate fiber geometry in development since 1998 [48, 49, 66–71, 77]. These edge detection models include snakes active contours [48, 66], ridge detection utilizing Hessian operators [67, 68], standard filtering and thresholding [70–72], or variations of watershed algorithms [49, 69]. Many of these efforts allow the user to adjust specific parameters for the segmentation or post hoc evaluation to merge or separate fibers that were poorly segmented as well as a filtering step to remove small objects or those which do not match certain shape requirements [45, 49, 69, 71]. Once fibers are segmented, simple shape parameters like cross-sectional area or ferret diameter are used to assess pathology or immunostaining intensity of specific markers to perform fiber type analysis. Kastenschmidt et al. utilized a supervised approach to allow the user to annotate examples of fibers vs non-fibers to perform binary classification [45]. This approach first performs thresholding and filtering to segment objects within the image and then extracts shape features which are used by the support vector machine to classify the objects. Similarly, Encarnacion-Rivera and colleagues performed pre-segmentation convolutional filtering, which then allows users to select training pixels for a random forest classifier to segment the edges of the fibers, and utilizes mean fluorescent histograms to select thresholds classifying muscle fibers [73].

#### Other

There are very few examples of computational pathology in other MSK fields, including wound healing or dermal complications of connective tissue diseases. Zhange et al utilized a murine bone fracture healing model stained with OrangeG/Alcian Blue to study how an automated model compares to hand drawn annotations [78]. After training the model on the colors associated with newly formed cartilage, newly formed bone, new fibrous tissue, bone marrow and new osteoblastic area; the authors implemented custom area-based adjustments (e.g., fills holes in cartilage to include the lacuna of the chondrocytes, change bone marrow between 30,000-200,000 μm2 to fibrous tissue). This algorithm produced areas that correlated very well with hand annotations (ICCs 0.97-0.99), however it was only evaluated on 5 tissue sections. We recently used unsupervised SLIC super pixel over segmentation and feature extraction to study wound healing and build a decision tree model which classified primary granulation tissue, secondary granulation tissue, and chondrogenic tissue in a murine model of tibial implant surgery [46]. Due to the limited sample size of the study, there were not enough images to reserve an internal test set for model validation. However, we worked with an expert pathologist to evaluate the accuracy of the model, and we found that the model predictions aligned well with the pathologist-scored outcomes. In systemic sclerosis (SSc), skin fibrosis is a key indicator of disease progression, and Masson trichrome is used to stain dense extracellular matrix, like collagens to show fibrotic activity. Correia et al. obtained biopsies stained with Masson trichrome from patients with SSc and utilized the pretrained weights of the feature encoder from the AlexNet DL architecture [6] to extract image features. PCA was then used to summarize these features into a single summary score which correlated well with other validated histologic scores (modified Rankin) and clinical outcomes [47]. These examples highlight the need and value of implementing computational histopathology approaches to other MSK conditions.

## Conclusion

Throughout the evolution of histological analysis, pathologists have improved their workflow and diagnostic accuracy by adopting the digitization of microscopic and histologic images [79]. Computational pathology and WSI have brought on a new era of computer-assisted analytical software [7], and the development of novel computational tools for image analysis has highlighted the utility of these procedures. However, these tools have also underscored the need for standardized protocols throughout the entire pipeline (from specimen collection to imaging) to permit the generalizability of datasets and approaches. Despite these obvious challenges, computational pathology will continue to evolve, and the fields of musculoskeletal health should be positioned to capitalized on these new analytical tools.

## Data Availability

All data and material is available upon request to the corresponding author.

## Abbreviations

MSK: Musculoskeletal
ML: Machine learning
DL: Deep learning
NN: Neural metwork
DNNs: Deep neural networks
WSI: Whole slide imaging
AI: Artificial intelligence
RGB: Red, green, blue
PCA: Principal component analysis
UMAP: Uniform Manifold Approximation and Projection
CNNs: Convolutional Neural Networks
SLIC: Simple linear iterative clustering
OA: Osteoarthritis
H&E: Hematoxylin and eosin
LEDs: Light emitting diode
CCD: Charged couple device
CMOS: Complementary metal-oxide-semiconductor
PMT: Photomultiplier tube
DAB: 3,3′-diaminobenzidine
FDA: Food and Drug Administration
ECM: Extracellular matrix
SSc: Systemic sclerosis
